# Low incidence of recurrence and chronic pain after groin hernia repair in adolescents: a systematic review and meta-analysis

**DOI:** 10.1007/s00423-023-02947-9

**Published:** 2023-05-26

**Authors:** Hugin Reistrup, Kristoffer Andresen, Jacob Rosenberg

**Affiliations:** Center for Perioperative Optimization, Department of Surgery, Herlev Hospital, University of Copenhagen, Copenhagen, Denmark

**Keywords:** Adolescents, groin hernia, inguinal hernia, recurrence, chronic pain, meta-analysis

## Abstract

**Purpose:**

The best operative management of groin hernia in adolescents is uncertain. The aim of this systematic review was to assess recurrence and chronic pain after mesh versus non-mesh repair for groin hernia in adolescents.

**Methods:**

A systematic search was done in PubMed, EMBASE, and Cochrane CENTRAL in May 2022 for studies reporting postoperative chronic pain (≥6 months) or recurrence after groin hernia repair in adolescents aged 10–17 years. We included randomized controlled trials and observational studies on primary unilateral or bilateral groin hernia repair. Risk of bias was assessed with the Cochrane risk-of-bias tool and Newcastle-Ottawa Scale. Meta-analysis of the incidence of recurrence was conducted. This review is reported according to PRISMA guideline.

**Results:**

A total of 21 studies including 3,816 adolescents with groin hernias were included comprising two randomized controlled trials, six prospective, and 13 retrospective cohort studies. For non-mesh repairs, the weighted mean incidence proportion of recurrence was 1.6% (95% CI 0.6–2.5) after 2,167 open repairs and 1.9% (95% CI 1.1–2.8) after 1,033 laparoscopic repairs. For mesh repairs, it was 0.6% (95% CI 0.0–1.4) after 406 open repairs while there were no recurrences after 347 laparoscopic repairs (95% CI 0.0–0.6). Across all surgical techniques, the rate of chronic pain after 1,153 repairs ranged from 0 to 11%. Follow-up time varied and was reported in various ways.

**Conclusion:**

The incidences of recurrence after groin hernia repair in adolescents were low for both open and laparoscopic mesh and non-mesh repairs. Rates of postoperative chronic pain were low.

**Trial registration:**

PROSPERO: CRD42022130554.

**Supplementary Information:**

The online version contains supplementary material available at 10.1007/s00423-023-02947-9.

## Introduction

Groin hernia repair is a common surgical intervention in both pediatric and adult populations [1]. The best surgical management of groin hernias in adolescents is an area of uncertainty as these patients fall between the pediatric and adult populations. Among adolescents, there is a great physical variation since some patients will be fully grown while others will not, raising the question when an adolescent should be surgically treated as either a child or an adult. Mesh is routinely used in hernia repair in adults, but the use of mesh might be problematic in children due to concerns about placing a foreign body in growing tissue and concerns of living 70+ years with a foreign body.

At present, the surgical approach for adolescents is determined by the personal preferences of the surgeon in collaboration with the patient and parents. Operative methods include open or laparoscopic repair and use of mesh or sutures only. However, the preferred methods can depend on whether the surgeon is mostly handling pediatric or adult patients [2]. In an international survey among pediatric surgeons, 83% preferred an open approach while 17% preferred a laparoscopic approach [3]. Mesh is preferred in adults as it lowers recurrence rates [4, 5], but a common postoperative complication is chronic pain occurring in rates as high as 8–15%, depending on definition and method of assessment [6–10]. Young adult males are at greater risk of chronic pain than older males following inguinal hernia repair, although the role of the mesh is uncertain [11]. There are indications from a recent retrospective study that mesh in late adolescence (18–21 years) may increase chronic postoperative pain [12], but the area needs more evidence.

The aim of this study was to assess recurrence and chronic pain after mesh versus non-mesh groin hernia repair in adolescents.

## Materials and methods

This systematic review was conducted according to the Preferred Reporting Items for Systematic Reviews and Meta-Analyses (PRISMA) guideline [13]. The protocol was registered at PROSPERO prior to completion of screening and prior to initiation of data extraction (registration number: CRD42022130554) [14]. Approval from an ethics committee was not needed for this study.

### Eligibility criteria

#### Population

The participants of the included studies were males or females aged 10–17 years.

#### Intervention

The intervention of interest was primary unilateral or bilateral groin hernia repair. Both open and laparoscopic repairs were included.

#### Outcomes

The primary outcomes were hernia recurrence and postoperative chronic pain. Recurrence had to be determined by physical examination or diagnostic imaging, hence, studies with only self-reported recurrence were excluded. If a study did not explicitly outline the method for assessing recurrence, but the study design and study characteristics indicated that recurrence was reliably assessed, the study was included. Reoperation for recurrence was used as a proxy for recurrence. There was no minimum follow-up time on recurrence for inclusion in this review. Postoperative chronic pain was defined as pain ≥6 months after surgery, hence, studies reporting pain had to have a minimum follow-up of six months [15]. At least one of the primary outcomes (recurrence or chronic pain) had to be reported for a study to be included. The secondary outcome was postoperative complications. The definition of age was of specific importance as this review aimed to include adolescents. According to the World Health Organization, the definition of adolescents is 10–19-year-olds [16]. The hernia literature generally defines adults from 18 years and above. Therefore, we defined adolescents as 10–17-year-olds.

#### Study design

Study types included in this review were randomized controlled trials (RCT) and observational studies with ≥5 participants. Studies reported in English, Danish, Swedish, or Norwegian were included.

### Information sources

The systematic search was conducted in PubMed, EMBASE, and Cochrane CENTRAL. Authors were contacted if reporting of data was unclear. Specifically, authors were contacted if data for the age group of 10–17-year-olds were not presented. The search strategy was developed in cooperation with a professional research librarian. The search was last conducted on 9 May 2022 in all three databases.

### Search strategy

The search was developed for PubMed and afterwards adapted to EMBASE and Cochrane CENTRAL. The systematic search was divided into three blocks: hernia AND age AND (pain OR recurrence). In EMBASE, the limitation “exclude medline journals” was used with confidence that all MEDLINE-registered articles were found in PubMed. The search strategy can be accessed in the PROSPERO protocol (registration number: CRD42022130554). The full search strategy in PubMed was:

(((((("Hernia, Inguinal"[Mesh]) OR "Hernia, Femoral"[Mesh])) OR (((((inguinal) OR femoral) OR groin)) AND hernia))) AND ((((((((((((((((("Adolescent"[Mesh]) OR adoles*) OR preadoles*) OR pre*adoles*)) OR teen*) OR juvenil*) OR (((("Puberty"[Mesh]) OR pube*) OR prepube*) OR pre*pube*)) OR (("Young Adult"[Mesh]) OR young*)) OR youth*) OR ((underage*) OR under*age*)) OR (((("Pediatrics"[Mesh]) OR pediatric*) OR paediatric*) OR peadiatric*)) OR ((("Child"[Mesh]) OR child*) OR children*)) OR ((kid) OR kids)) OR (("Minors"[Mesh]) OR minor*)) OR boy*) OR girl*)) AND ((((("Pain"[Mesh]) OR "Chronic Pain"[Mesh]) OR pain*)) OR (((((((recurren*) OR "Recurrence"[Mesh])) OR ((reoperat*) OR "Reoperation"[Mesh])) OR re-operat*) OR relapse*) OR revision*))

Records were imported to EndNote (version X7.8) for removal of duplicates. Screening was done in Rayyan [17]. Title and abstract were screened independently by two authors according to predefined eligibility criteria. Full text articles were screened by one author, and all suggested included articles where further screened by another author. Doubts on eligibility for inclusion were discussed within the author group. A snowball search was conducted to find possible relevant articles for inclusion from the reference lists of the included articles [18].

### Data collection

Data extraction was done by one author and entered into predefined tables in an Excel sheet (Microsoft Excel for Mac, version 15.32), and all data were validated at least twice by the same author. Doubts regarding data extraction were discussed within the author group. Extracted variables included study design, year, outcomes, number and age of participants, hernia characteristics, type of repair, pain, recurrence, follow-up, intra- and postoperative complications, analgesia, and surgeon experience*.*

### Study risk of bias assessment

The risk of bias in RCTs was assessed with a revised Cochrane risk-of-bias tool for randomized trials (RoB 2) [19]. The risk of bias in observational studies was assessed with the Newcastle-Ottawa Scale [20]. The higher the number of stars a study gets assigned with the Newcastle-Ottawa Scale, the lower the risk of bias. In general, a study can get a maximum of nine stars, indicating low risk of bias. For observational studies, the risk of bias was assessed for the two primary outcomes (recurrence and chronic pain) separately (Table 1 and Table S1). If a study only included either an exposed (mesh) or non-exposed (non-mesh) cohort, the study could be awarded a maximum of eight stars. The risk of bias assessment was done independently by two authors. Discrepancies in the assessment were discussed within the author group until a consensus was reached.

**Table 1:** Study characteristics

Author	Year	Study type	Adolescents, no.	Hernias, no.	Male, %	Age, years	Hernia type	Repair method		Follow-up time, months		NOS score	
								Mesh application (mesh type)	Approach	Adolescent cohort	Entire cohort	Recurrence	Chronic pain^l^
Pogorelić et al. [26]	2022	Prosp	51	57	73	13 (12–16)^c^	Inguinal	Non-mesh	Lap	44 (35–51)^c^	NA	5	NA
Cao et al. [27]	2022	Retro	167	-	-	10–14 ^f^	Inguinal	Non-mesh	Lap	6^h^	NA	5	NA
Muntean et al. [28]	2021	Retro	7	7	-	10–16 ^f^	Femoral	Non-mesh	Open	-	1 (0–30)^g^	4	NA
Taylor et al. [29]	2020	Retro	256	267	84	15 (13–16)^c^	Inguinal	Non-mesh	Open	68 (35–119)^c^	NA	5	NA
Gibbons et al. [30]	2020	Retro	135	166	64	14 (12–16)^c^	Inguinal	Non-mesh	Lap	30 (22–77)^c^	NA	4	NA
Chu et al.^a^ [31]	2020	Retro	349	372^i^	75^b^	15 (±1)^e^	Inguinal	Non-mesh	Lap	-	65 (±25)^e^	7	NA
			95	95		16 (±1)^e^		Mesh (biologic)	Open				
Lee [32]	2018	Retro	244	255	66	13 (±2)^d^	Inguinal	Non-mesh	Lap	41 (±5)^d^	NA	4	2
Criss et al. [33]	2018	Retro	64	74	94	16 (±2)^e^	Inguinal	Non-mesh	Both	38 (3–84)^c^	NA	5	4
Cui et al. [34]	2018	RCT	506	-	76	15 (±2)^d^	Groin	Mesh (synthetic)	Both	16 (5–34)^i^	NA	NA	NA
Pogorelić et al. [35]	2017	Retro	668	-	-	10–17^f^	Inguinal	Non-mesh	Open	-	168 (12–288)^k^	5	NA
Gasior et al. [36]	2015	Retro	210	-	88	15 (±2)^e^	Inguinal	Non-mesh	Open	59 (19–160)^g^	NA	4	4
Shen et al.^a^ [37]	2014	RCT	50	50	86	15 (±2)^e^	Inguinal	Non-mesh	Open	33 (±3)^e^	NA	NA	NA
			50	50	84	15 (±2)^e^		Mesh (biologic)		33 (±3)^e^			
Bisgaard et al.^a^ [38]	2014	Prosp	441	441	80^b^	8–17^f^	Inguinal	Non-mesh	Open	12^h^	NA	6	NA
			86	86		8–17^f^		Mesh (-)	Both				
Saad et al. [39]	2011	Prosp	187	219	-	9–18^f^	Inguinal	Non-mesh	Open	-	12–120^f^	3	NA
Liu et al. [40]	2011	Prosp	14	-	-	13–18^f^	Inguinal	Non-mesh	Lap	-	40 (2–110)^j^	2	NA
Zendejas et al. [41]	2010	Retro	36	39	-	10–18^f^	Inguinal	Non-mesh	Open	-	591 (567–626)^g^	4	NA
Ein et al. [42]	2006	Retro	143	153	83	13–18^f^	Inguinal	Non-mesh	Open	-	-	4	NA
Taqvi et al. [43]	2006	Prosp	16	-	-	10–12^f^	Inguinal	Non-mesh	Open	-	9^h^	4	NA
Huang et al. [44]	2005	Prosp	8	-	-	11–20^f^	Groin	Mesh (synthetic)	Open	-	5–41^f^	3	NA
Mayagoitia [45]	2004	Retro	8	-	88	15 (11–17)^g^	Inguinal	Mesh (synthetic)	Open	5 (4–5)^g^	NA	5	4
Lund et al. [46]	1987	Retro	25	25	96	15–19^f^	Groin	Non-mesh	Open	-	-	3	NA

^a^: study is presented twice in the table to present data for the mesh and non-mesh groups separately, ^b^: as reported for the whole adolescent cohort including both mesh and non-mesh repairs, ^c^: median (interquartile range), ^d^: weighted mean (standard deviation), ^e^: mean (standard deviation), ^f^: range, ^g^: median (range), ^h^: maximum follow-up time, ^i^: presuming that all bilateral repairs were done laparoscopically (not reported), ^j^: weighted mean (range), ^k^: mean (range), ^l^: pain ≥6 months postoperatively, NOS: Newcastle-Ottawa Scale [20], Retro: retrospective cohort study, Prosp: prospective cohort study, RCT: randomized controlled trial, Lap: laparoscopic, -: not reported, NA: not applicable

### Data analysis

For meta-analysis, data on recurrence were analyzed with OpenMeta[Analyst] [21]. Forest plots and weighted mean incidence proportions of recurrence were produced. When meta-analysis was not feasible, data were presented descriptively. A binary random-effects model (DerSimonian and Laird [22]) with 95% confidence intervals was applied. When the lower limit of the confidence interval was negative in meta-analysis, it was reported as zero. A correction factor of 0.5 was used for outcomes with zero events. Sensitivity analysis was conducted on follow-up time, sample size omitting studies with less than 100 participants, and by omitting repairs that included femoral hernia. For quantifying heterogeneity across studies, the *I*^*2*^ statistic was applied. For chronic pain, meta-analysis was not feasible due to methodological heterogeneity between studies and results were therefore presented descriptively. If a study only reported the number of adolescents with hernia repairs, but did not report the specific number of repairs (e.g. if patients had bilateral repairs), the number of adolescents with hernia repairs would be used as a surrogate measure for the number of hernia repairs knowing that this would likely underestimate the total number of repairs.

### Certainty assessment

The Grading of Recommendations, Assessment, Development, and Evaluations (GRADE) framework [23] and the GRADEpro software [24] were used to assess the certainty of evidence for the primary outcome of recurrence. To guide the assessment, the GRADE Handbook [23] and a guide on the application of GRADE for prognostic studies were used [25].

## Results

**Fig. 1:**
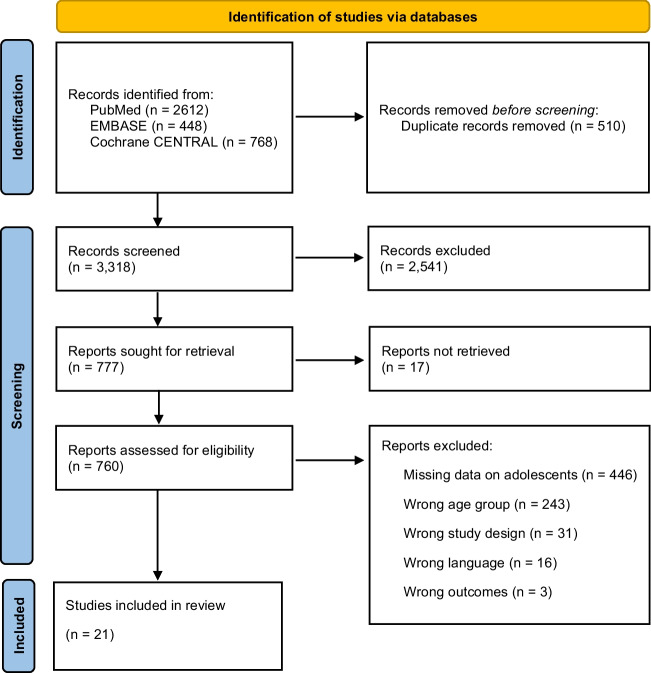
PRISMA diagram [13] of study inclusion

Twenty-one studies were included in this review [26–46]. See Fig. 1 for flow diagram depicting the screening process. We contacted the corresponding authors of 334 studies to obtain data relevant for this review when data were not reported in the individual studies. The authors of two studies [35, 45] supplied unpublished data that enabled inclusion in this review.

Two randomized controlled trials [34, 37], six prospective cohort studies [26, 38–40, 43, 44] and 13 retrospective cohort studies [27–33, 35, 36, 41, 42, 45, 46] with data on a total of 3,816 adolescent patients with 3,953 groin hernia repairs were included. Overall, there were 3,200 non-mesh and 753 mesh repairs. Of these, 2,573 were open and 1,380 were laparoscopic repairs. Fifteen studies [26–30, 32, 33, 35, 36, 39–43, 46] reported non-mesh repairs, three studies [34, 44, 45] reported mesh repairs, and three studies [31, 37, 38] reported both non-mesh and mesh repairs. Six studies [29, 33, 36, 39, 40, 42] on a total of 937 repairs explicitly reported the surgeons as pediatric surgeons, while none explicitly reported the surgeons as adult surgeons. The included studies reported data on adolescent patients in various ways. Seven studies [26, 29, 30, 32, 34, 36, 37] reported data on adolescents only. In 14 studies [27, 28, 31, 33, 35, 38–46], data on adolescents were extracted from a population with a wider age-range than defined in this review. For these studies, the reporting on relevant data other than our primary outcomes for the adolescent population were sparse. Follow-up time varied and was reported for the adolescent population in 11 studies [26, 27, 29, 30, 32–34, 36–38, 45] and was reported in various ways. In eight studies [28, 31, 35, 39–41, 43, 44], follow-up time for the whole study cohort was reported, but not for the adolescent subpopulation. Two studies [42, 46] did not report follow-up time. See Table 1 for study characteristics.

**Fig. 2:**
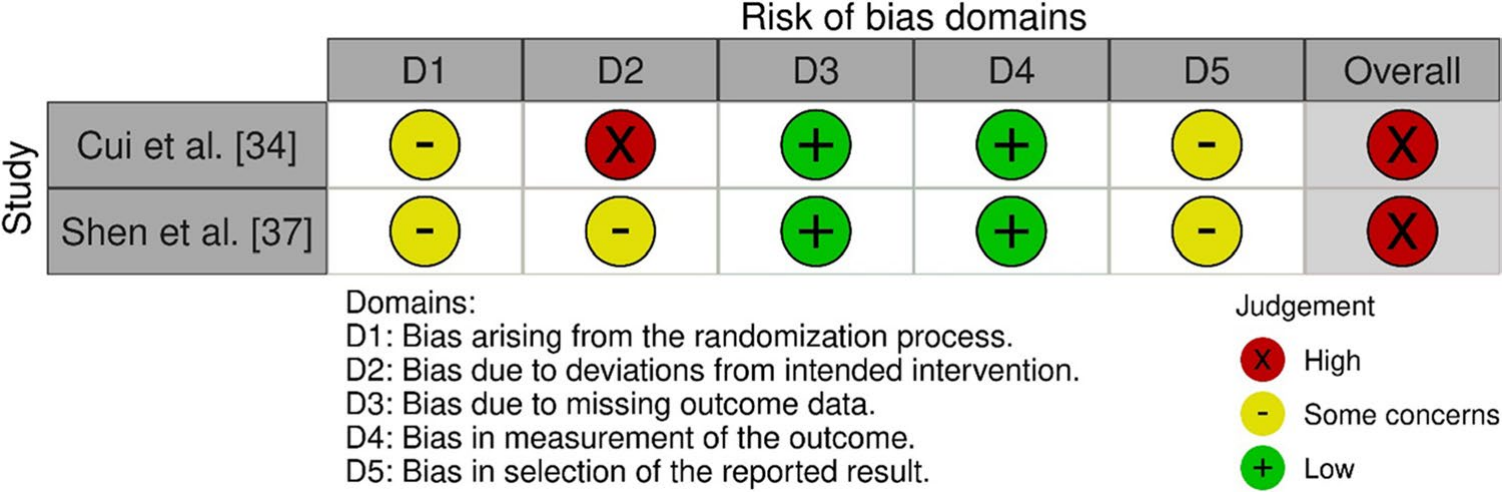
Risk of bias assessment for randomized controlled trials [19, 47]

Separate risk of bias assessments were performed for each of the two primary outcomes of this review. The assessments of two randomized controlled trials [34, 37] were similar across all domains for both recurrence and chronic pain, respectively (Fig. 2 [47]). For cohort studies, the Newcastle-Ottawa Scale was used. For recurrence, six prospective [26, 38–40, 43, 44] and 13 retrospective [27–33, 35, 36, 41, 42, 45, 46] cohort studies were assessed resulting in a median of four stars (range 2–7) (Table 1 and Table S1). For chronic pain, four retrospective [32, 33, 36, 45] cohort studies were assessed resulting in a median of four stars (range 2–4) (Table 1 and Table S1).

### Recurrence

All 21 included studies [26–46] reported recurrence rates. Recurrence was assessed in various ways: clinical assessment [26, 29, 35, 37, 43–45], clinical assessment and additional methods (phone interview, chart review, or imaging) [31–33, 39, 40, 42], questionnaires [30, 36], questionnaires and chart review [41, 46], chart review only [28], ultrasound [27], and registry data [38]. In one study [34], where the method of assessment of recurrence was not reported, the study design was an RCT, and the authors reported that patients were followed for three years giving sufficient confidence in the assessment method to include the study in this review. However, the corresponding author did not respond to our enquiry. In two studies [30, 36] with self-reported recurrence, all patients with recurrence were either reoperated or the recurrence was later verified by a doctor. Follow-up time varied and was reported in various ways (Table 1).

### Open non-mesh repair

**Fig. 3:**
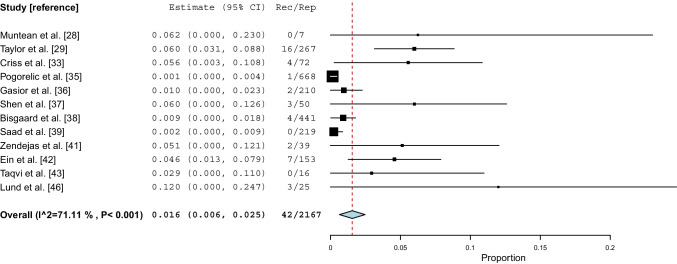
Incidence proportions of recurrence after open non-mesh groin hernia repair. *CI: confidence interval, Rec: recurrences, Rep: repairs, P: P value*

Twelve studies [28, 29, 33, 35–39, 41–43, 46] on a total of 2,167 repairs reported recurrence rates for open non-mesh repairs from 0 to 12%. Meta-analysis showed a weighted mean incidence proportion of recurrence of 1.6% (95% CI 0.6–2.5, *I*^*2*^=71%, P<0.001) (Fig. 3). The certainty of evidence was considered moderate (Table S2). When excluding two studies where data on inguinal and femoral hernia were not reported separately [46] or when only including femoral hernias [28], a sensitivity analysis resulted in a similar incidence proportion of recurrence. Furthermore, sensitivity analysis on studies with a minimum follow-up of 12 months [35–39, 41] and sensitivity analysis omitting studies with <100 participants [28, 33, 37, 41, 43, 46] also showed a similar incidence proportion of recurrence.

### Laparoscopic non-mesh repair

**Fig. 4:**
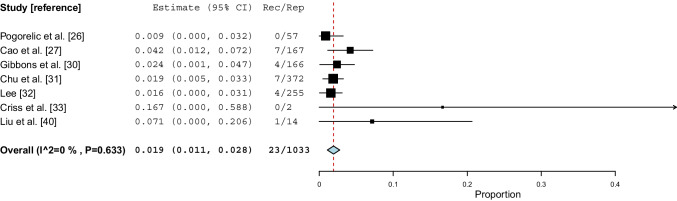
Incidence proportions of recurrence after laparoscopic non-mesh groin hernia repair. *CI: confidence interval, Rec: recurrences, Rep: repairs, P: P value*

Seven studies [26, 27, 30–33, 40] on a total of 1,033 repairs reported recurrence rates for laparoscopic non-mesh repairs from 0 to 7.1%. All studies reported data on inguinal hernia repair only. Meta-analysis showed a weighted mean incidence proportion of recurrence of 1.9% (95% CI 1.1–2.8, *I*^*2*^=0%, P=0.633) (Fig. 4). The certainty of evidence was considered moderate (Table S2). Sensitivity analysis on studies with a minimum follow-up of 6 months [26, 27, 30–32, 40] and sensitivity analysis omitting studies with <100 participants [26, 33, 40] showed a similar incidence proportion of recurrence.

### Open mesh repair

**Fig. 5:**
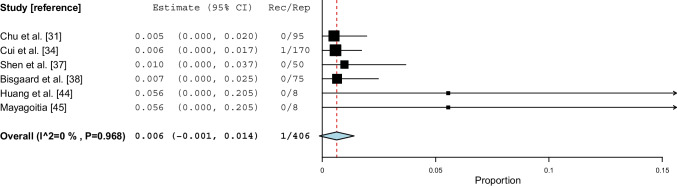
Incidence proportions of recurrence after open mesh groin hernia repair. *CI: confidence interval, Rec: recurrences, Rep: repairs, P: P value*

Six studies [31, 34, 37, 38, 44, 45] on a total of 406 repairs reported recurrence rates for open mesh repairs. One study [34] accounted for 42% of the repairs. Five studies [31, 37, 38, 44, 45] on a total of 236 repairs reported no recurrences, and one study [34] on 170 repairs reported one recurrence. The weighted mean incidence proportion of recurrence was 0.6% (95% CI 0.0–1.4, *I*^*2*^=0%, P=0.968) (Fig. 5). The certainty of evidence was considered moderate (Table S2). Three studies [34, 44, 45] used synthetic mesh, two studies [31, 37] used biologic mesh, and one study [38] did not report the type of mesh used.

### Laparoscopic mesh repair

Two studies [34, 38] on a total of 347 repairs reported recurrence rates for laparoscopic mesh repairs. One study [34] accounted for 97% of the repairs. There were no recurrences (95% CI 0.0–0.6, *I*^*2*^=0%, P=0.486). The certainty of evidence was considered moderate (Table S2).

### Chronic pain

Six studies [32–34, 36, 37, 45] on a total of 1,153 hernia repairs reported pain six months or more postoperatively. For non-mesh repairs, two studies [36, 37] reported rates of chronic pain for open repairs of 7% (15/210) and 0% (0/50), respectively, and one study [32] reported a rate of 0% (0/255) for laparoscopic repairs. For mesh repairs, one study [34] reported a rate of chronic pain for open repairs of 2.9% (5/170), and two studies [37, 45] both reported a rate of 0% (0/50 and 0/8, respectively). One study [34] on laparoscopic mesh repair reported a rate of chronic pain of 1.5% (5/336). Two studies [33, 34] compared open with laparoscopic repairs. One study [34] did not find a significant difference in chronic pain when comparing the two groups, and the other study [33] did not report data on chronic pain separately for the open and laparoscopic repair groups for the adolescent subgroup. Follow-up time varied and was reported in various ways (Fig. 1).

Tools for the assessment of pain varied. One study [32] used the Visual Analog Scale (VAS) and stated that there was no occurrence of chronic pain, but they did not define the term chronic pain or report when pain was measured. One study [37] measured postoperative pain with the VAS two hours postoperatively, but also stated that no patients had chronic pain without defining the term or tool used for later pain assessment. One study [34] used the Numeric Rating Scale reporting chronic pain in 2% of adolescents, also not stating a definition of chronic pain. One study [36] reporting chronic pain in 7% of adolescents used a telephone questionnaire with yes or no questions including a question on residual pain from the operation. In another questionnaire study [33] using yes or no questions that were either e-mailed or phoned, 11% of adolescents answered yes to the question: *do you have any pain associated with your hernia repair?* And in one study [45] reporting no occurrence of chronic pain, an interview was conducted at follow-up, but the study did not state the specific method or questions used for the evaluation of chronic pain. Due to heterogeneity of the included studies, meta-analysis on chronic pain was not performed.

### Postoperative complications

Eight studies [26, 28–30, 32–34, 37] on a total of 1,363 adolescent patients with 1,432 hernia repairs reported postoperative complications other than pain or recurrence for adolescents. Across all studies, the definition and reporting of postoperative complications varied.

One study [29] on open non-mesh repair reported a rate of postoperative complications of 4.1%. Postoperative complications were defined as surgical site infection, symptomatic hematoma, symptomatic hydrocele, postoperative pain requiring emergency department visit or pain management referral, or significant postoperative nausea/vomiting requiring admission. In one study [33], where 98% of the adolescents had an open non-mesh repair and 2% had a laparoscopic non-mesh repair, a 30-day surgical site infection rate of 3% was reported. In another study [30] on laparoscopic non-mesh repair, wound infection occurred in 0.7% and stitch abscess in 1.5% of patients. A study [32] on laparoscopic non-mesh repair reported no wound infections, but 1.2% developed a hematoma and 0.8% a seroma. In one study [37] on open repair with a non-mesh and a mesh group, 11.6% of the adolescents in the non-mesh and 14.3% in the mesh group developed a hydrocele postoperatively. Two studies [26, 28] on a total of 64 groin hernia repairs stated that there were no postoperative complications.

## Discussion

This systematic review demonstrates that most data in the literature on recurrence after groin hernia repair in adolescents are on open non-mesh repair. Overall, incidences of recurrence were low across all surgical approaches including open and laparoscopic mesh and non-mesh repairs. The rate of chronic pain was also low across all surgical techniques, though results should be interpreted with caution due to data scarcity and study heterogeneity. Data on postoperative complications other than recurrence and chronic pain were sparsely reported but seem to be low.

Most of the data in the literature on recurrence after groin hernia repair concerns inguinal hernia. In children under the age of 12 years, the recurrence rate was 1.4% (137/9,993) after non-mesh repair (96% were open repairs) [48]. In adults, a Cochrane review found a recurrence rate of 1.8% (52/2,834) in the mesh repair group, compared with 4% (110/2,741) in the non-mesh repair group [4]. This systematic review found lower recurrence rates after both open and laparoscopic mesh and non-mesh groin hernia repair among adolescents compared with the results for adults in the Cochrane review [4]. There may be many reasons for this, including differences in follow-up between studies and likely better postoperative tissue healing in children compared with adults.

As with recurrence, most of the literature on postoperative chronic pain after groin hernia repair also concerns inguinal hernia. Overall, the rate of chronic pain is lower in children and adolescents compared with the rate of up to 8–15% reported in adults [6–10]. The prevalence of chronic pain was 5.1% after a follow-up time of 6–48 months in children aged six months to 12 years at the time of open non-mesh repair [49]. The prevalence of chronic pain was 13.5% after an observation period of 14–18 years in patients who had undergone open non-mesh repair before the age of 5 years [50]. Despite data scarcity, this systematic review indicates that rates of postoperative chronic pain in adolescents are similar compared to younger children and substantially lower than in adult hernia repair regardless of operative technique.

This systematic review has several strengths. The review was reported according to PRISMA guideline [13], and a protocol was uploaded on PROSPERO [14] prior to completion of screening and prior to initiation of data extraction, securing transparency. The search was developed with a professional research librarian and performed in several databases supplemented by a snowball search. The search strategy was broad to confidently search all available literature for a population whose age-range can be difficult to isolate in literature searches. All authors with available contact information were contacted if the reporting of data was unclear or to retrieve unpublished data if relevant, which was often due to studies not reporting data on the specific age group targeted by this review. The assessment of bias was conducted by two independent researchers and doubts were discussed within the author group. To ensure that postoperative pain was reported correctly as chronic pain, a minimum follow-up time of 6 months after the repair was set as a criterion for inclusion in this review.

Some limitations of this systematic review must also be mentioned. We were unable to retrieve 17 of the 777 articles for full-text screening, hence, some articles fulfilling our inclusion criteria might have been missed. There was a possibility of language bias as only studies in English, Danish, Swedish, and Norwegian were included. The Newcastle-Ottawa Scale was used for assessing the risk of bias in observational studies, though the latest version of the Cochrane Handbook recommends using the Risk Of Bias In Non-randomized Studies of Interventions (ROBINS-I) tool [51]. Underestimation of recurrence rates is probable as we accepted studies using reoperation for recurrence as a proxy for recurrence even though it has been shown to underestimate recurrence rates by up to 40% [52]. Furthermore, recurrence was evaluated at various time points and in some studies using uncertain assessment methods. Also, several studies measured recurrence using a combination of initial clinical assessment and later questionnaires with self-reported recurrence. The reporting of pain was done with varying pain assessment tools and at various time points. Lastly, follow-up times were reported in various ways across all studies and in several studies not specifically for the adolescent population inhibiting comparison of studies on follow-up time.

## Conclusion

In conclusion, our systematic review found that incidences of recurrence in adolescents after both open and laparoscopic mesh and non-mesh repairs were low. Rates of postoperative chronic pain were also low across all surgical approaches, but data scarcity hinders firm conclusions on this outcome.

### Supplementary Information

ESM 1 (DOCX 23.6 KB)

ESM 2 (DOCX 25.1 KB)
