# Supplementary material for: Low incidence of recurrence and chronic pain after groin hernia repair in adolescents: a systematic review and meta-analysis
Source: Langenbecks Arch Surg. 2023 May 26;408(1):211. doi: 10.1007/s00423-023-02947-9 (PMC10220125; doi:10.1007/s00423-023-02947-9)
Supplement: Supplementary file 1 — ESM 1 (DOCX 23.6 KB) [file 423_2023_2947_MOESM1_ESM.docx]

**Supplementary Table S1** Risk of bias assessment of observational studies using the Newcastle-Ottawa Scale (NOS)

| **Outcome** | | **Selection** | | | |  | **Comparability** |  | **Outcome** | | | **Total stars/score** |
| --- | --- | --- | --- | --- | --- | --- | --- | --- | --- | --- | --- | --- |
|  | **Author** | **Representativeness of the exposed (mesh) cohort** | **Selection of the non-exposed (non-mesh) cohort** | **Ascertainment of exposure** | **Demonstration that outcome of interest was not present at start of study** |  | **Comparability of cohorts on the basis of the design or analysis** |  | **Assessment of outcome** | **Was follow-up long enough for outcomes to occur** | **Adequacy of follow up of cohorts** |  |
| Recurrence | |  |  |  |  |  |  |  |  |  |  |  |
|  | Pogorelić et al. [26] | - | * | * | * |  | - |  | * | * | - | 5 |
|  | Cao et al. [27] | - | * | * | * |  | * |  | * | - | - | 5 |
|  | Muntean et al. [28] | - | * | * | * |  | - |  | * | - | - | 4 |
|  | Taylor et al. [29] | - | * | * | * |  | - |  | * | * | - | 5 |
|  | Gibbons et al. [30] | - | * | * | * |  | - |  | - | * | - | 4 |
|  | Chu et al. [31] | * | * | * | * |  | - |  | * | * | * | 7 |
|  | Lee [32] | - | - | * | * |  | - |  | - | * | * | 4 |
|  | Criss et al. [33] | - | * | * | * |  | - |  | * | * | - | 5 |
|  | Pogorelić et al. [35] | - | * | * | * |  | - |  | * | - | * | 5 |
|  | Gasior et al. [36] | - | * | * | * |  | - |  | - | * | - | 4 |
|  | Bisgaard et al. [38] | * | * | * | * |  | - |  | * | - | * | 6 |
|  | Saad et al. [39] | - | * | * | * |  | - |  | - | - | - | 3 |
|  | Liu et al. [40] | - | - | * | * |  | - |  | - | - | - | 2 |
|  | Zendejas et al. [41] | - | * | * | * |  | * |  | - | - | - | 4 |
|  | Ein et al. [42] | - | - | * | * |  | - |  | * | - | * | 4 |
|  | Taqvi et al. [43] | - | * | * | * |  | - |  | * | - | - | 4 |
|  | Huang et al. [44] | - | - | * | * |  | - |  | * | - | - | 3 |
|  | Mayagoitia [45] | * | - | * | * |  | - |  | * | * | - | 5 |
|  | Lund et al. [46] | - | * | * | * |  | - |  | - | - | - | 3 |
| Chronic pain | |  |  |  |  |  |  |  |  |  |  |  |
|  | Lee [32] | - | - | * | - |  | - |  | - | * | - | 2 |
|  | Criss et al. [33] | - | * | * | - |  | - |  | * | * | - | 4 |
|  | Gasior et al. [36] | - | * | * | - |  | - |  | * | * | - | 4 |
|  | Mayagoitia [45] | * | * | * | - |  | - |  | - | * | - | 4 |
