# Supplementary material for: Low incidence of recurrence and chronic pain after groin hernia repair in adolescents: a systematic review and meta-analysis
Source: Langenbecks Arch Surg. 2023 May 26;408(1):211. doi: 10.1007/s00423-023-02947-9 (PMC10220125; doi:10.1007/s00423-023-02947-9)
Supplement: Supplementary file 2 — ESM 2 (DOCX 25.1 KB) [file 423_2023_2947_MOESM2_ESM.docx]

**Supplementary Table S2** The Grading of Recommendations, Assessment, Development, and Evaluations (GRADE) assessment of recurrence

| **Number of studies [reference]** | **Certainty assessment** | | | | | | **Effect** | | | **Certainty** |
| --- | --- | --- | --- | --- | --- | --- | --- | --- | --- | --- |
|  | **Study design** ^a^ | **Risk of bias** | **Inconsistency** | **Indirectness** | **Imprecision** | **Other considerations** | **Number of events** | **Number of individuals** | **Rate (95% CI)** |  |
| Open non-mesh repair | | | | | | | | | | |
| 12 [28,29,33,35–39,41–43,46] | Observational studies | Serious | Not serious | Not serious | Not serious | None | 42 | 2167 | Event rate 1.6 per 100 (0.6 to 2.5) | ⨁⨁⨁◯ Moderate |
| Laparoscopic non-mesh repair | | | | | | | | | | |
| 7 [26,27,30–33,40] | Observational studies | Serious | Not serious | Not serious | Not serious | None | 23 | 1033 | Event rate 1.9 per 100 (1.1 to 2.8) | ⨁⨁⨁◯ Moderate |
| Open mesh repair | | | | | | | | | | |
| 6 [31,34,37,38,44,45] | Observational studies | Serious | Not serious | Not serious | Not serious | None | 1 | 406 | Event rate 0.6 per 100 (0.0 to 1.4) | ⨁⨁⨁◯ Moderate |
| Laparoscopic mesh repair | | | | | | | | | | |
| 2 [34,38] | Randomized trials | Serious | Not serious | Not serious | Not serious | None | 0 | 347 | Event rate 0.2 per 100 (0.0 to 0.6) | ⨁⨁⨁◯ Moderate |

^a^: study design from which the majority of participants derive, CI: confidence interval
